# Whole-Genome Resequencing of Twenty *Branchiostoma belcheri* Individuals Provides a Brand-New Variant Dataset for *Branchiostoma*

**DOI:** 10.1155/2020/3697342

**Published:** 2020-01-24

**Authors:** Changwei Bi, Na Lu, Tingyu Han, Zhen Huang, J.-Y. Chen, Chunpeng He, Zuhong Lu

**Affiliations:** ^1^State Key Laboratory of Bioelectronics, School of Biological Science and Medical Engineering, Southeast University, Nanjing, China; ^2^The Public Service Platform for Industrialization Development Technology of Marine Biological Medicine and Product of State Oceanic Administration, College of Life Sciences, Fujian Normal University, Fuzhou, Fujian, China; ^3^Key Laboratory of Special Marine Bio-Resources Sustainable Utilization of Fujian Province, Fuzhou, Fujian, China; ^4^Nanjing Institute of Paleontology and Geology, Chinese Academy of Sciences, Nanjing, China

## Abstract

As the extant representatives of the basal chordate lineage, amphioxi (including the genera *Branchiostoma*, *Asymmetron* and *Epigonichthys*) play important roles in tracing the state of chordate ancestry. Previous studies have reported that members of the *Branchiostoma* species have similar morphological phenotypic characteristics, but in contrast, there are high levels of genetic polymorphisms in the populations. Here, we resequenced 20 *Branchiostomabelcheri* genomes to an average depth of approximately 12.5X using the Illumina HiSeq 2000 platform. In this study, over 52 million variations (~12% of the total genome) were detected in the *B. belcheri* population, and an average of 12.8 million variations (~3% of the total genome) were detected in each individual, confirming that *Branchiostoma* is one of the most genetically diverse species sequenced to date. Demographic inference analysis highlighted the role of historical global temperature in the long-term population dynamics of *Branchiostoma*, and revealed a population expansion at the Greenlandian stage of the current geological epoch. We detected 594 Single nucleotide polymorphism and 148 Indels in the *Branchiostoma* mitochondrial genome, and further analyzed their genetic mutations. A recent study found that the epithelial cells of the digestive tract in *Branchiostoma* can directly phagocytize food particles and convert them into absorbable nontoxic nutrients using powerful digestive and immune gene groups. In this study, we predicted all potential mutations in intracellular digestion-associated genes. The results showed that most “probably damaging” mutations were related to rare variants (MAF<0.05) involved in strengthening or weakening the intracellular digestive capacity of *Branchiostoma*. Due to the extremely high number of polymorphisms in the *Branchiostoma* genome, our analysis with a depth of approximately 12.5X can only be considered a preliminary analysis. However, the novel variant dataset provided here is a valuable resource for further investigation of phagocytic intracellular digestion in *Branchiostoma* and determination of the phenotypic and genotypic features of *Branchiostoma*.

## 1. Introduction

Vertebrates, urochordates, and cephalochordates (also known as lancelets or amphioxi), belonging to the phylum Chordata, evolved from a common ancestor that lived about 520–550 million years ago [1, 2]. Most chordates evolved into a variety of vertebrates under two rounds of whole-genome duplication (2R-WGD); however, the genome of amphioxi remained intact without any WGD events [2]. For these reasons, amphioxi are considered to be intermediate between vertebrates and invertebrates, and thus, are widely used as a model organism to study the evolution of invertebrate and the origin of vertebrates [1–6]. Previous studies have found that amphioxi are extremely genetically diverse animals with high population heterozygosity [2, 3, 7–9]. However, amphioxi still maintain extreme similarity in their phenotypic characteristics, despite the high rate of genetic polymorphisms. The recently released whole-genome sequencing of* B. belcheri* provides a valuable reference and strategy for resequencing of the *Branchiostoma* species [3]. Further, the variant dataset provided in this study can shed further light on the genomic features of *Branchiostoma* and the origin of vertebrates.

Mitochondria are double-membrane-bound organelles in the cytoplasm of most eukaryotic cells, with the exception of mature mammalian red blood cells. The core function of mitochondria is to convert the chemical energy derived from food into adenosine triphosphate (ATP), which can be directly used by cells. Although most DNA is packaged in the nucleus, mitochondria also have a small amount of their own DNA. The human mitochondrial (mt) DNA contains 37 genes, including 13 protein-coding genes, 22 transfer RNAs, and two ribosomal RNAs, all of which are essential for normal mt function [10]. Previous studies have demonstrated that mutations in mt genomes may incite dysfunctions or other unpredictable changes [11–13]. The mt 12S ribosomal RNA gene encodes a protein that regulates insulin sensitivity and metabolic homeostasis, and mutations of this gene have been found to cause hearing loss [14, 15]. The mt tRNA-Lys gene is involved in the assembly of proteins to carry out oxidative phosphorylation; mutations of this gene can result in multiple mt deficiencies and associated disorders [16, 17]. The mt ATPase 6 protein forms one part (subunit) of a large enzyme called ATP synthase; its mutations may affect the final step of oxidative phosphorylation in mitochondria [18]. Previous studies have demonstrated that the gene organization of the *Branchiostoma* mt genome is identical to that of humans [19–21]. The current study aims to identify genetic mutations in the whole mt genome of *B. belcheri* and decipher the genetic background of mt-related functions in *Branchiostoma*.

Animals take advantage of mitochondria to generate energy to survive; energy is primarily generated from phagocytizing and digesting food particles through intracellular or extracellular digestion [22, 23]. Previous studies have found that phagocytic intracellular digestion is the evolutionary cornerstone of digestive and immune mechanisms of multicellular animals [24–26]. *Branchiostoma* is a perfect model organism to facilitate analysis of the evolution of immune and digestive mechanisms of animals as both intracellular and extracellular digestion can be observed in the *Branchiostoma* digestive process [27]. However, previous studies of *Branchiostoma* have only focused on the evolution of vertebrate immune mechanisms, rather than the original digestive function of *Branchiostoma* [28–30]. Recently, He et al. observed both phagocytic intracellular and extracellular digestion in *Branchiostoma* by transmission electron microscopy and scanning electron microscopy [31]. They detected a number of phagocytic intracellular digestion-associated genes in *Branchiostoma* epithelial cells, including digestive or hydrolytic genes, immune reaction-associated genes, and typical immune genes, which can directly phagocytize food particles, such as algal cells. In order to investigate the genetic features of these intracellular digestion-associated genes, it is crucial to understand whether mutations in these genes affect their functions in intracellular digestion.

In this study, we employed a resequencing strategy to generate 20 *B. belcheri* genomes with ~12.5X depth using the Illumina Hiseq 2000 system. These sequences were then mapped to the *B. belcheri* (v.18h27) genome to generate genotype calls. We explored the genome-wide genetic divergence of the *Branchiostoma* population and of each individual. Demographic inference revealed that the effective population size of *Branchiostoma* may have suffered from various degrees of reduction during the four major glaciations in the Quaternary, but these were followed by a remarkable population expansion during the interglacial Greenlandian stage of the current geological epoch. Notably, we identified all specific nonsynonymous variants within phagocytic intracellular digestion-associated genes, and further predicted their functional effects in 20 sequenced *Branchiostoma* individuals. The variant dataset presented here is a valuable resource for further investigation of phagocytic intracellular digestion in *Branchiostoma*, and for the investigation of phenotypic and genotypic features of *Branchiostoma*.

## 2. Materials and Methods

### 2.1. Sample Preparation, DNA Extraction, and Sequencing

Twenty *B. belcheri* individuals, 10 male and 10 female, were obtained from Zhanjiang, Guangdong province, for whole-genome resequencing. Genomic DNA of the 20 individuals was extracted separately using a QIAamp® DNA mini kit (Qiagen, Germany) following the standard manufacturer's protocol. The purity and concentration of total DNA were determined with a NanoDrop spectrophotometer (NanoDrop, Wilmington, DE). DNA integrity was assessed by agarose gel electrophoresis. Briefly, the DNA sample was fragmented using a Covaris ultrasonic processor (Covaris, USA) to a size of ~350 bp, then the fragmented DNA was end repaired, “A”-tailed, and ligated with the full-length adaptor for Illumina sequencing with further PCR amplification. The concentrations of the constructed libraries were initially measured and diluted to 1 ng/μl by Qubit®2.0 (Life technologies, USA). Then, an Agilent Bioanalyzer 2100 system (Agilent, USA) was used to check the insert size of the libraries. To ensure the quality of these constructed libraries, the SYBR green qRT-PCR protocol was used with a Kapa Probe Fast qPCR kit (Kapa Biosystems, USA) to accurately dose the effective concentrations of the libraries. Finally, these libraries were sequenced on the Illumina HiSeq 2000 platform (Illumina, USA) by the Novogene Bioinformatics Institute, Beijing, China.

### 2.2. Filtering and Mapping of Reads

To ensure the sequencing reads were reliable and did not contain low-quality paired reads, the sequencing raw reads were pre-processed with a series of quality control (QC) steps [32]. The following QC criteria were applied to remove low-quality reads:

1. Removal of reads with more than 10% unidentified nucleotides (N).
2. Removal of reads containing more than 50% of bases with a Phred score ≤ 5.
3. Removal of putative PCR duplicate reads generated by PCR amplification using SAMtools [33, 34].

After removing low-quality reads, the clean paired-end reads were mapped to the *B. belcheri* v.18h27 reference genome and the mt genome (GenBank accession: NC_004537) using BWA-MEM (v0.7.15) with the following parameters: -M, -k 19 [35]. The BWA-MEM algorithm performs local alignment, which may produce multiple primary alignments for different parts of a query sequence, especially for highly heterozygous genomes. Therefore, we used the option –M to flag shorter split hits as secondary, and then filtered them from the generated SAM files using sambamba with the following parameters: -F “not (secondary_alignment or Supplementary)” −p −l 9 [36]. The remaining mapped reads were then sorted and converted into BAM format files using SAMTOOLS and were then marked as PCR duplicates using the GATK MarkDuplicates module (ver. 4.0.2.1) [37]. In addition, the Qualimap bamqc tool was used to estimate genome coverage and depth of mapped reads on the reference genome [38].

### 2.3. Detection and Filtration of Genetic Variations

**Figure 1:**
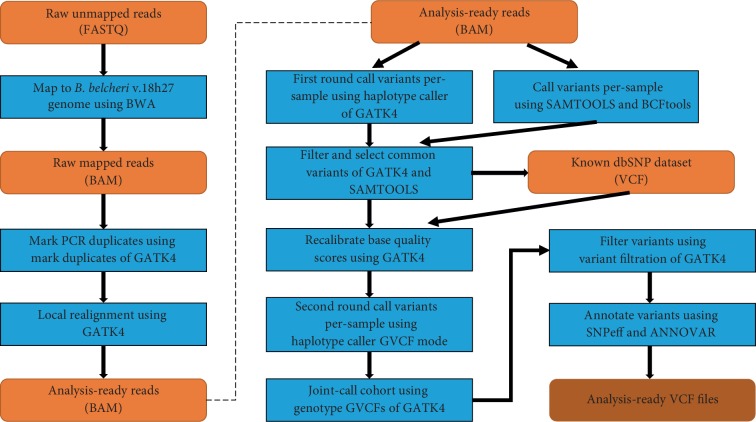
Workflow for SNP & InDel discovery in our study using GATK4. This workflow includes sequence alignment, variation calling, filtration, and annotation. The orange rounded rectangles indicate the generated files and the light blue rectangles indicate the processing programs.

The quality scores of the individual base calls only reflect the confidence in the specified nucleotide; however, the actual probabilities of erroneous base calls may be weakly correlated with their quality scores [39]. To standardize the quality scores across sequencing runs and libraries, we performed empirical quality score recalibration using GATK4. Since there was no known dbSNP dataset for *Branchiostoma*, we needed to first define an SNP dataset as the known dbSNP dataset that could then be used in the subsequent steps (Figure 1).

The generation of the known dbSNP dataset was performed as follows. First, we applied the GATK3 RealignerTargetCreator and IndelRealigner modules to reduce the false-positive SNPs where alignment error occurred across overlapping reads. Second, the GATK4 HaplotypeCaller module and SAMTOOLS mpileup command were used to detect SNPs and Indels with the bam files generated from step 1. Third, the same variations shared by both tools were selected using the GATK4 SelectVariants module, and then strict parameters (for SNPs and Indels: QD < 10.0||MQ < 50.0||FS > 10.0||MQRankSum < −5.0||ReadPosRankSum < −8.0) were used to filter these selected variations using the GATK4 VariantFiltration module. Finally, steps 2 and 3 above were repeated until the generated variations converged; the conserved variations were defined as the known dbSNP dataset.

Thereafter, we used the GATK4 BaseRecalibrator and ApplyBQSR modules to generate recalibrated bam files for each individual. Then, we used the GATK4 HaplotypeCaller module with the GVCF model to detect variations from the recalibrated bam files. The generated GVCF files from the 20 sequenced individuals (BB_Male1-10, BB_Female1-10) were then merged to generate a raw population genotype file using the CombineGVCFs and GenotypeGVCFs modules in GATK4. Further, we applied the GATK4 SelectVariants module to split SNPs and Indels from the generated population genotype file (VCF format). Then, we applied the hard filter module “VariantFiltration” to exclude potential false-positive variant calls with the following parameters: SNPs (QD<2.0 || MQ<40.0 || FS>60.0 || MQRankSum<−12.5 || ReadPosRankSum<−8.0 || QUAL<30); Indels (QD<2.0 || FS>200.0 || ReadPosRankSum<−20.0 || QUAL<30).

In order to detect and compare SNPs and Indels in each individual, we used the GATK4 GenotypeGVCFs module to process the GVCF format files from the 21 individuals (BB_Male1-10, BB_Female1-10, and SRR1174914). The generated variation call format (VCF) files were then filtered under the above conditions, except that the DP value was set as DP > 2 || DP < 25, according to a previous study [40]. Then, we compared variations between each two sequenced individuals using the BCFtools isec module (ver. 1.3.1). The transitions and transversions for each individual were calculated by VCFtools (0.1.15) with the parameter “–FILTER-summary” [41].

### 2.4. Annotation of Genetic Variations

In order to annotate the genetic variations detected in the *Branchiostoma* genome, we obtained the genome annotation file in gff3 format and the transcript and protein files in fasta format for the *B. belcheri* genome (ver.18h27) from the NCBI Genome Database. These retrieved files contained the detailed genomic coordinates of gene, coding DNA sequence (CDS), exon, intron, and untranslated region (UTR) for each annotated gene. Using these genome annotation files, we applied both ANNOVAR [42] and SNPeff [43] to classify the detected SNPs/Indels into exonic regions, splicing sites (2 bp within a splicing junction), ncRNA (overlapping a transcript without coding annotation), 5′ and 3′ UTRs, intronic regions, upstream and downstream regions (1 kb region upstream or downstream of a transcription start or end site), and intergenic regions. The SNPs/Indels located in exonic regions might result in variations at the protein level. The SNPs/Indels identified in UTRs might cause a gain or loss of start/stop codons, thereby affecting translation efficiency. The SNPs/Indels in upstream/downstream regions (1 kb away from the transcription start site or the transcription end site) might influence transcription factor's binding affinity, thus altering gene expression at the RNA level.

### 2.5. Estimation of Demographic History

We used the pairwise sequentially Markovian coalescent (PSMC) software to infer the demographic history of *B. belcheri* [44]. This software uses the distribution of heterozygous sites across the genome sequence and a PSMC model that defines the hidden Markov model. We first used the program “fq2psmcfa,” provided by PSMC software, to transform the consensus sequence into the required fasta-like input format. The program “psmc” was then used to infer the population size history with the following parameters: time interval = 6 + 29×2; numbers of iterations = 25; mutation rate per generation = 1×10^−8^; generation time = 3. The time interval and the number of iterations were chosen manually according to suggestions given in the PSMC software (https://github.com/lh3/psmc), and the mutation rate and generation time were obtained from a previous *Branchiostoma* genome study [3].

### 2.6. Prediction of Functional Effect of SNP-Associated Genes in Diverticulum Epithelial Cells

Phagocytic intracellular digestion is a very important mechanism in *Branchiostoma*. A recent study showed that *Branchiostoma* diverticulum epithelial cells express different genes when they are starved or sated. The expressed sequence tags (ESTs) were obtained from the study by He C. et al. (GenBank accession number: LIBEST_028542) [31]. The local program BlastN was then applied to annotate and identify which genes were highly expressed in diverticulum epithelial cells according to their EST clusters [45]. Then, we divided them into different genetic functional types using the results generated by BlastN, including digestive or hydrolytic genes, immune reaction-related genes, and typical immune genes. Finally, we used the online program PolyPhen-2 to predict the functional and structural effects of nonsynonymous SNP (nsSNP)-associated genes which are highly expressed in diverticulum epithelial cells [46]. The batch query of the PolyPhen-2 program was obtained by using the annotation information from ANNOVAR; all protein sequences of these SNP-associated genes were used as another input file for the program. Then, these nonsynonymous sites were classified into different categories based on pairs of 5%/10% false positive rate (FPR) thresholds; the categories included: probably damaging (FPR ≤ 0.05), possibly damaging (0.05 < FPR ≤ 0.1), and benign (FPR > 0.1). If no prediction could be made due to no homologous regions within the individual, then the outcome was reported as “unknown”.

## 3. Results and Discussion

### 3.1. Genome Sequencing and Mapping

Previous studies have shown that the polymorphism rates of the *Branchiostoma* genome are much higher than those of other animals; yet, *Branchiostoma* have retained their ancestral body plan and morphology since the Cambrian period [2–4]. In order to investigate genetic variants in the entire *Branchiostoma* genome, we examined 20 *Branchiostoma* individuals, which were captured at Zhanjiang, Guangdong province. Then, we extracted genomic DNA from their muscular tissues and performed DNA sequencing with the Illumina HiSeq/MiSeq platform to generate 150 bp paired-end reads. After a series of QC processes, we obtained a total of 42,180,039 high-quality clean reads (99.43% of raw reads), which covered approximately 127 Gbp. The clean reads were then mapped back to the *B. belcheri* v.18h27 reference genome and mt genome with BWA-MEM (v0.7.15) using the –M, −k 19 parameters. An average of 95.85% of reads from the 20 sequenced individuals could be mapped to the reference genome (Table 1, [Supplementary-material supplementary-material-1]). The average effective genome-wide coverage and depth for our 20 sequenced individuals were 86.26% and 12.49X, respectively, while the coverage and depth for the high-depth sequencing individuals (SRR1174914) were 89.69% and 42.3X, respectively.

### 3.2. Detection of Genetic Variations in *B. belcheri* Population

**Figure 2:**
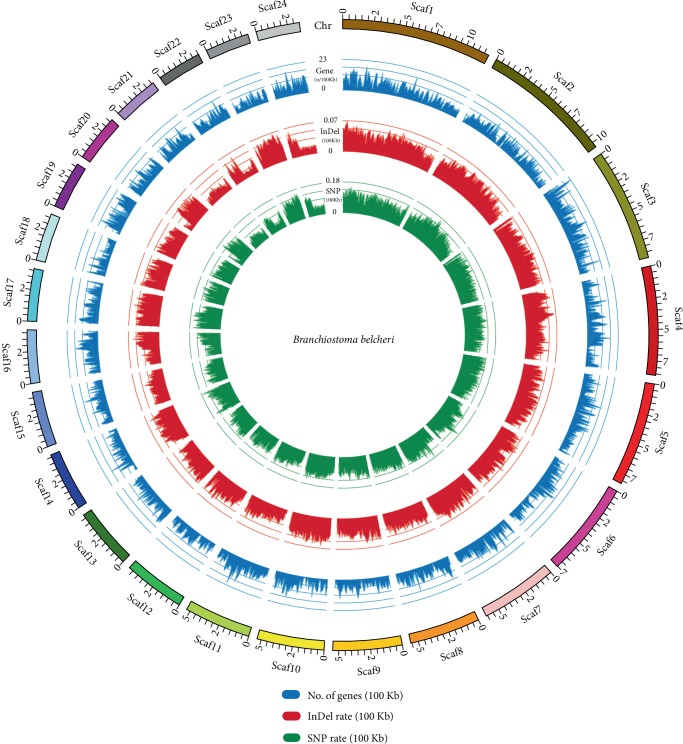
The SNP & InDel and gene density of representative 24 scaffolds. The numbers of SNPs, Indels, and genes per 100 kb are shown as red, green, and blue, respectively.

The generated alignment bam format files for each individual were further processed with SAMTOOLS and GATK4. After rigorous variation filtration, a total of 52,130,473 sites (12.23% of total genome) in the *B. belcheri* v.18h27 nuclear genome, including 37,589,099 SNPs and 14,541,374 Indels, were identified to be mutated in one or more individuals (Table 2). In order to visualize the genomic variations, we chose the largest 24 scaffolds as representatives to show the distribution of gene numbers, SNPs, and Indels in the genome (Figure 2). Figure 2 shows that the *Branchiostoma* genome has an extremely high number of polymorphisms. Among the high-quality SNPs identified in the *B. belcheri* population, 18,795,212 were transitions, 12,867,914 were transversions (Ts/Tv = 1.46), and the remaining 5,925,973 sites had more than two variants. We also identified the Ts/Tv ratio in exonic regions; the ratio was 1.91, which is higher than that in the whole genome. Previous studies, particularly from the 1000 Genomes Project, have revealed that the Ts/Tv ratio for the whole human genome is 2–2.1, while for human exomes it is 2.8–3.0, and for novel SNPs it is around 1.5 [47, 48]. The Ts/Tv ratio for novel SNPs is lower than the whole genome and exomes, probably because novel SNPs tend to be nsSNPs rather than synonymous SNPs [49]. Therefore, we can infer that most mutations in *B. belcheri* occurred recently with a high mutation per generation.

**Figure 3:**
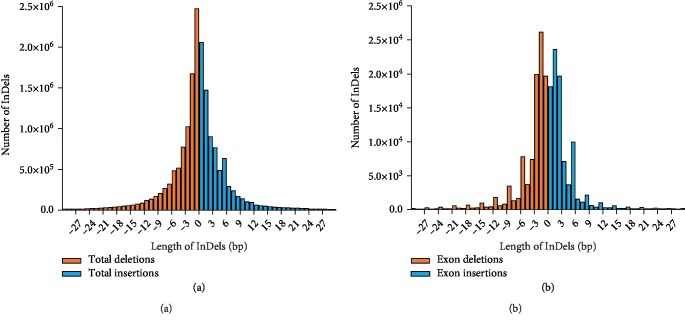
Distribution of insertion and deletion lengths in the *B. belcheri* genome. Numbers of insertions and deletions in the whole genome (a) and in exonic regions (b). Insertions and deletions are shown in orange and light blue bars, respectively.

To evaluate the functional consequences of the variants in CDS regions, the total CDS variations were divided into 4,412,877 SNPs and 224,658 Indels (Table 2). The CDS SNPs were further divided into 1,467,863 nonsynonymous, 2,818,189 synonymous, 11,487 stop codon gained, 1,275 stop codon loss, and 114,063 unknown SNPs. Similarly, 134,185 Indels within exons can cause frameshifting (non-3x-bp length), 78,739 Indels cannot cause frameshifting (3x bp length), 5,820 and 280 Indels can cause the gain or loss of a stop codon, respectively, and 5,634 Indels belong to unknown regions (Table 2). As shown in Figure 3, the largest proportion of Indels in the total genome was single base pairs, while in exons, the largest proportion was double base pairs. Variations in nsSNPs and frameshift Indels could result in amino acid changes, thus affecting function at the protein level, while variations in stop codon gain or loss regions might affect translation efficiency. Unknown regions were defined as any exonic mutations identified in transcripts with the premature stop codon. Future studies must investigate the effect of these potential variations on gene function.

**Figure 4:**
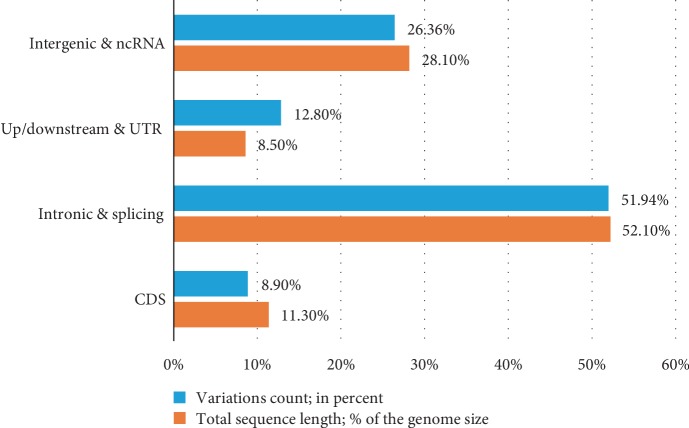
The fraction of variations mapped to the four genomic regions. The genomic regions and count of variations are shown in orange and light blue bars, respectively.

Aside from the variations identified in the exonic region, we found that most variations (27,053,764 sites; 51.9% of total variations) were located in intronic regions rather than the expected intergenic regions; this may be because of the larger number of introns found in the *B. belcheri* v.18h27 genome (Figure 4). As described by Huang et al. [3], the proportion of intronic regions and CDS in the *Branchiostoma* genome is much higher than in other vertebrates and some invertebrates. In the current study, the proportion of CDS was found to be higher than the variations in the genome, while the proportions of up/downstream (1kb) and UTRs were lower than the variations in the genome (Figure 4). These proportions were consistent with other species, probably because CDS must maintain high conservatism to perform their relevant functions, and noncoding or UTRs do not have this requirement. Further, 21,669 variations (5,702 SNPs and 15,967 Indels) were found in the splicing region; this is defined as a variant within 2 bp in the intron that is close to an exon. Variations in the splicing region might alter pre-RNA splicing, thus generating several new introns or resulting in the loss of native exons in mature RNA.

### 3.3. Detection of Genetic Variations in *B. belcheri* Individuals

In order to further investigate the variations in *Branchiostoma* individuals, we also genotyped SNPs and Indels in each *B. belcheri* individual. As shown in Table 3, an average of 12,772,354 variations, including 9,924,229 SNPs and 2,848,125 Indels, were identified in our 20 sequenced *B. belcheri* genomes. Among these detected variations, the average proportion of heterozygous sites among the whole genome was 1.66%. According to neutral theory, the high level of heterozygosity in the *Branchiostoma* genome is a result of a large effective population size or an increased mutation rate [39, 50]. Additionally, we also detected the Ts/Tv ratios for each *Branchiostoma* individual and obtained a steady ratio of 1.31, which is slightly smaller than the ratio in the *Branchiostoma* population ([Supplementary-material supplementary-material-1]).

**Figure 5:**
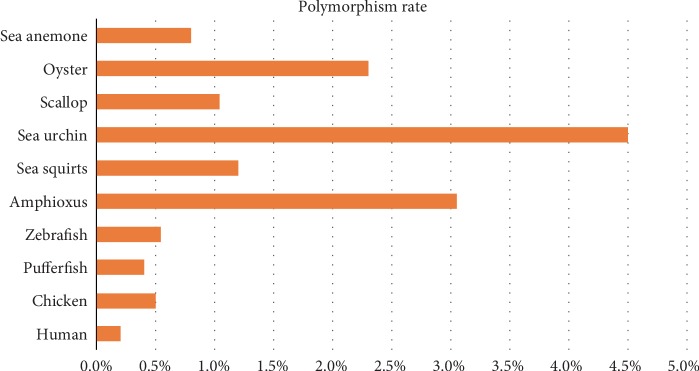
The polymorphism rates of ten different animals. A total of ten species were selected in this study, including four vertebrates (human, chicken, pufferfish, and zebrafish), a cephalochordate (amphioxus), a urochordate (sea squirts), an echinoderm (sea urchin), two lophotrochozoans (scallop, wild oyster), and a cnidarian (sea anemone) [48, 51–58].

The polymorphism rates of our sequenced *Branchiostoma* individuals ranged from 2.67% with the lowest sequencing depth to 3.17% with the highest depth (mean of 3.04%) (Table 1, Table 3, [Supplementary-material supplementary-material-1]). This suggests that low-depth sequencing data causes loss of some polymorphisms. When we used the high-depth sequencing data (43.3X) to identify variations in *Branchiostoma*, the polymorphism rate increased to 3.69% (Table 1, [Supplementary-material supplementary-material-1]), which is still smaller than that of previously published reports (4% for *B. floridae* and 5.37% for *B. belcheri*) [2, 3]. The difference in the polymorphism rate between *B. floridae* and *B. belcheri* is likely because both species have undergone a long period of independent evolution since they diverged from the most recent common ancestor approximately 100 million years ago [6]. A previous study by Huang *et al.* detected SNPs and Indels using custom Perl scripts [3]; this study did not filter the generated variations, likely leading to overestimation of polymorphisms in the final variation dataset. As shown in Figure 5, the polymorphism rate of *Branchiostoma* was extremely high when compared to other animals with available sequencing data. The polymorphism rates of 10 selected species are reported to vary from 0.14% in humans, 0.4% in pufferfish, 0.54% in zebrafish, 0.6% in chickens, and 0.8% in sea anemone to up to 4-5% in an echinoderm sea urchin [48, 51–55]. The polymorphism levels of Lophotrochozoa (oysters and scallops), Echinodermata (sea urchins), Cephalochordata (amphioxi), and Urochordata (sea squirts) are over 10 variations per kilobases [56–58].

To further investigate the genetic feature of the *Branchiostoma* genome, we compared the detected variations in our 20 sequenced *Branchiostoma* individuals. Among all detected variations, the number of variations shared among all 20 individuals was 455,768 (0.11% of the whole genome), including 409,210 SNPs and 46,558 Indels. This suggests that these genomic sites in the *B. belcheri* v.18h27 reference genome are probably sequence errors. A total of 36,513,048 variations (72.53% of all variations; 27,738,992 SNPs and 8,774,056 Indels) were shared by at least two individuals, and the remaining variations were confined to a single individual. As shown in [Supplementary-material supplementary-material-1], the polymorphism rates between each two *Branchiostoma* individuals were almost identical, as were the polymorphism rates of our samples compared to the reference genome, indicating that the *Branchiostoma* species has a high level of genetic mutations, even among individuals living in the same sea area.

We also applied ANNOVAR and SNPeff to annotate the variations identified in each *Branchiostoma* individual to investigate their characteristics. Based on the gene annotations from the *B. belcheri* reference genome, we found that most of the variations in our sequenced individuals were located in intronic regions (55.69%; 7,112,803) and intergenic regions (21.36%; 2,727,701), while the remaining variations were located in exonic regions (9.71%; 1,240,040), 1 kb up/downstream to a gene (8.99%; 1,148,319), and UTRs (4.22%; 539,323); only 4,167 variations were found in splicing sites ([Supplementary-material supplementary-material-1]). The proportion of variations in each genomic feature was consistent with the above population analyses.

In order to investigate the differences between high depth and low depth data, we compared the variations in exons, splicing regions, up/downstream regions, UTRs, introns, and intergenic regions. As shown in [Supplementary-material supplementary-material-1], a total of 2,954,903 variations were lost in our low-depth sequencing data, including 248,055 variations in exonic regions, 1,297 variations in splicing sites, 262,348 variations in sites 1 kb up/downstream to a gene, 88,611 variations in UTRs, 1,641,041 variations in intronic regions, and 713,552 variations in intergenic regions. The greatest variation loss was observed in intronic and intergenic regions.

### 3.4. Inference of Demographic History

**Figure 6:**
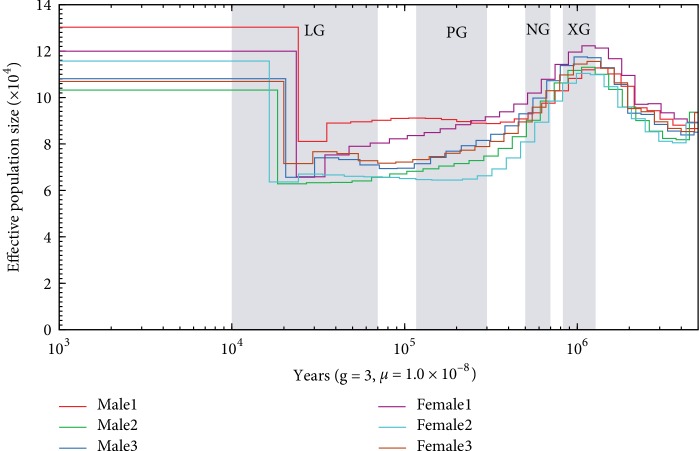
The inferred demographic history of *B. belcheri*. Reconstruction of the historical patterns of effective population size for six *B. belcheri* genomes using the PSMC method. The number of years per generation (g) and the neutral mutation rate per generation (*μ*) were assumed to be 3 years and 1.0 × 10^−8^, respectively. Four major glaciations in Quaternary, including the Xixiabangma Glaciation (XG, 0.8–1.17 Ma), Naynayxungla Glaciation (NG, 0.5–0.72 Ma), Penultimate Glaciation (PG, 0.13–0.3 Ma), and Last Glaciation (LG, 0.01–0.07 Ma) are shaded in gray.

In order to explore the ancestral demographic trajectories of *B. belcheri*, we estimated the changes in effective population size using the PSMC method [44]. The demographic history inferences are shown in Figure 6. We chose six *Branchiostoma* individuals, three males and three females, to represent the *B. belcheri* population. The inferred ancestral demographic trajectories were very similar for all analyzed *Branchiostoma* individuals across most of the species' history, suggesting cohesiveness of the species. As shown in Figure 6, all six selected *B. belcheri* individuals first experienced a remarkable population expansion during the Gelasian stage of the Pleistocene epoch (~1.80–2.58 Ma), which is the first epoch of the Quaternary Period. The effective population size of *B. belcheri* continued to increase in the early Calabrian stage of the Pleistocene epoch (~1.30–1.80), but suffered from a large reduction during the later period of the Calabrian stage (~0.7–1.30 Ma), when suitable climates were lost. This reduction lasted for much of the Chibanian stage of the Pleistocene epoch (~0.13–0.78 Ma). The *B. belcheri* population size was then very stable in the Tarantian stage from approximately 0.0117 Ma to 0.13 Ma. Subsequently, *B. belcheri* experienced a large-scale population increase in the Greenlandian stage of the current geological epoch, referred to as the Holocene epoch (0.0082–0.0117 Ma), when the glacial periods passed and the interglacial period arrived. The *B. belcheri* population has been relatively stable until more recent times.

The population fluctuations in the demographic history of *B. belcheri* may correspond to the different glacial periods during the Pleistocene epoch [59]. Previous studies have reconstructed the Quaternary climatic history of the Qinghai–Tibetan Plateau using glacial geologic data [60]. Four major glaciations, with average temperatures 2–6°C lower than the present temperatures, are recognized in the Quaternary, including the Xixiabangma Glaciation (XG; 0.8–1.17 Ma), Naynayxungla Glaciation (NG; 0.5–0.72 Ma), Guxiang Glaciation (PG; the Penultimate: 0.13–0.3 Ma), and the Baiyu Glaciation (LG; the Last: 0.01–0.07 Ma). As shown in Figure 6, the effective population size of *B. belcheri* suffered from reductions of various degrees during the first two glacial periods (XG and NG) and remained at a very low level in the other periods of the Quaternary, suggesting that the living environment of the ancient *Branchiostoma* population may have been susceptible to historical temperature. However, it should be noted that the estimation of effective population size over time largely depend on the parameters used in the PSMC software. As shown in [Supplementary-material supplementary-material-1], if we adopt a shorter generation time parameter (−g 2), the effective population size would increase in the first glacial period (XG) and decrease in the following two glacial periods (NG and PG). Thereafter, the effective population size would remain at a very low level during the early LG period, but would experience a remarkable population expansion during the later LG period. Additionally, the substrate and water quality of habitat can also influence the subsistence of *Branchiostoma;* this is probably the main reason for fluctuations in the ancient population. For example, *Branchiostoma* (productivity >60tons per year) in the Xiamen sea area were almost extinct due to the construction of the Gaoji sea walls (between Xiamen Gaoji and Jimei Peninsula).

### 3.5. Analysis of Genetic Divergence in B. belcheri Mitochondrial Genome

Mitochondria play a prominent role in the production of ATP and many other cellular metabolic tasks, such as regulation of the membrane potential, apoptosis-programmed cell death, certain heme synthesis reactions, steroid synthesis, and so on [61–63]. Additionally, the complete mt genome is also effectively used in molecular ecology, conservation and population genetics, and evolutionary biology [64, 65]. After mapping clean reads back to the *B. belcheri* mt genome, a total of 650 nucleotide positions, including 415 SNPs and 235 Indels, were found to be mutated in the *Branchiostoma* population (Table 2). The 415 SNPs consisted of 262 transitions (C-T and A-G) and 153 transversions (A-C, A-T, C-G, and G-T), with a Ts/Tv ratio of 1.72. Additionally, 321 of the 650 variations (49.38%) were identified within protein-coding genes, 232 variations were found in two rRNA genes (35.69%; 12S and 16S rRNA), 93 variations were distributed in tRNA genes, and only 4 variations were found in intergenic regions. We further analyzed the variations identified in mt protein-coding genes. As shown in Table 4, among the 13 mt protein-coding genes, three genes (*ND2*, *ND4L,* and *ND6*) did not show any variations, indicating that they were widely conserved during *Branchiostoma* evolution. Only one gene (*COX1*) showed neutral selection with a Ka/Ks ratio of exactly 1, and the other mt protein-coding genes exhibited purifying selection with Ka/Ks ratios less than 1. Nonsynonymous and frameshift mutations are destructive in the protein translation process, generating abnormal or nonfunctional proteins. In the *B. belcheri* mt genome, we identified 174 synonymous, 51 nonsynonymous, 81 frameshift, and 17 nonframeshift mutations. Additionally, 8 of the 22 mt tRNA genes (*tRNA-Arg, tRNA-Gln*, *tRNA-Glu*, *tRNA-Gly*, *tRNA-Lys*, *tRNA-Met*, *tRNA-Pro,* and *tRNA-Thr*) were extremely conservative without any variations, suggesting that the functions of these tRNA genes were highly conserved in *Branchiostoma* evolution. Mutations found in mt tRNAs can be responsible for diseases such as Mitochondrial encephalopathy, lactic acidosis, and stroke-like episodes (MELAS) and Myoclonic epilepsy with ragged-red fibers (MERRF) syndromes [12]. The variations identified in the *Branchiostoma* mt genome provide valuable information for the future study of *Branchiostoma* mt function and evolution.

### 3.6. Functional Annotation of Variations in Intracellular Digestion-Associated Genes

It is commonly accepted that mitochondria generate energy for cell survival from the digestion of food. Most heterotrophic unicellular organisms phagocytize and digest food particles directly via intracellular digestion [66], while most deuterostomes digest food using extracellular digestion; the latter involves breaking down large food particles into small, water-soluble absorbable molecules [23]. Since 1937, biologists have reported that, aside from general extracellular digestion, the diverticulum of *Branchiostoma* can directly phagocytize and digest food particles throughout all life stages [27]. A recent study by He C. et al. illustrated phagocytic intracellular digestion in *Branchiostoma* using a special tissue fixation method. They found many typical immune genes expressed in the epithelial cells of the *Branchiostoma* digestive tract [31]. In order to detect which genes play key roles in this phagocytic process, they constructed a full-length cDNA transcriptome library and sequenced the total RNA in the natural state of diverticulum epithelial cells using the Sanger method. In this study, we only focused on genes with nsSNPs that are highly expressed in phagocytic intracellular process.

In order to obtain accurate expression levels of phagocytic intracellular digestion-associated genes, the downloaded ESTs were first aligned to the total CDS of the *B. belcheri* genome to count the EST numbers of each gene using BlastN. As shown in Table 5, most ESTs belonged to three families, *cathepsin*, *ferritin*, and *trypsin*, which occupied 65.99% of the total tissue-specific (diverticulum phagocytic epithelial cells) genes. We then used the PolyPhen-2 program to predict the effects of nsSNPs in phagocytic intracellular digestion-associated genes, which predicts the effects of nsSNPs according to the genetic sequence and structural features of the ancestral allele with those of the derived allele. Among the identified nsSNPs, only two genes (*cathepsin D* and *Toll-interacting protein*) did not contain any “probably damaging” or “possibly damaging” mutations, whereas the others contained at least one “probably damaging” mutation; these mutations are likely to have effects on protein function or structure. In contrast, most of the genes with nsSNPs were determined to be “benign,” indicating that these nsSNPs do not alter protein function or structure. Three genes (*lysozyme*, *pancreatic lipase*-*like protein,* and *plasminogen*) contained too many “unknown” nsSNPs due to the lack of homologous regions in humans.

Further analysis of the PolyPhen-2 predicted results revealed that the nsSNP rates varied from a minimum of 0.12% in *Toll-interacting protein* to a maximum of 12.26% in *VCBP5* (Table 5). The nsSNP rates of typical immune genes expressed in diverticulum epithelial cells were higher than those of digestive, hydrolytic, and immune reaction-associated genes, suggesting that diverticulum epithelial cells of wild amphioxi require more functional alterations to immune genes to phagocytize and digest various food particles via intracellular digestion. Additionally, we also identified the nsSNPs in nine other immune genes and nine housekeeping genes that are expressed in the whole genome. As shown in Table S5, the nsSNP rates in the housekeeping genes were significantly lower than those of the immune genes, and there was only one “probably damaging” SNP in the housekeeping genes: *EF1A* (*elongation factor 1-alpha*). The high nsSNP rates in immune genes of wild amphioxi are probably due to the very complex environment in which they live and the presence of various microbial infections [5, 67].

In this study, we primarily focused on the “probably damaging” nsSNPs in phagocytic intracellular digestion-associated genes. The genes containing over 10 “probably damaging” mutations were *VCBP5*, *trypsin-like serine protease*, *chitotriosidase 1-like protein*, *arylsulfatase B*, *Cathepsin L*, *pancreatic lipase-likeprotein*, *VCBP4*, and* tetraspanin plasminogen*, while the others only contained a handful of “probably damaging” mutations (Table 5). As shown in [Supplementary-material supplementary-material-1], there were many patterns of alteration in amino acids; the most common trends were from valine (V) to methionine (M), serine (S) to cysteine (C), glycine (G) to arginine (R), or leucine (L) to phenylalanine (F). Minor allele frequency (MAF) refers to the frequency at which the second most common allele occurs in a given population; MAF can be used to differentiate common and rare variants in the population. Over 60% (138 out of 224) of “probably damaging” mutations were related to rare variants with MAF < 0.05, whereas the others were related to common variants with MAF>0.05 ([Supplementary-material supplementary-material-1]). Several studies have suggested that rare variants associated with risk of disease are preferentially situated in coding regions and have a greater influence on genomic function than the more common variants [68, 69]. Therefore, these genes with “probably damaging” mutations probably have entirely different functions or structures, which would have an influence on the function of phagocytic intracellular digestion-associated genes in *Branchiostoma*. For example, the “probably damaging” mutations in cathepsins affect intracellular protein catabolism functions in some way [70]. The “probably damaging” mutations in ferritins influence immune reaction abilities, because ferritins act as buffers to maintain the balance of iron in immune reactions, preventing the propagation of infections due to intracellular insufficient iron [71, 72]. The “probably damaging” mutations in typical immune genes, such as *VCBP*, *tetraspanin*, *alpha2-macroglobulin*, *bigdefensing*, and *Toll*-*interactingprotein*, would influence the immunocompetence of *Branchiostoma* [73–77].

## 4. Conclusion

In this study, we provide a variant database of the extant basal chordate *Branchiostoma* using a resequencing strategy on 20 *B. belcheri* individuals. Using the published *B. belcheri* genome as a reference, over 12% of genomic sites of the total genome were found to be mutated in at least one of the sequenced samples. An average of 12,772,354 variations (3.04% of total genome), including 9,924,229 SNPs and 2,848,125 Indels, were identified in each *B. belcheri* genome. Additionally, we found the Ts/Tv ratio of the whole *Branchiostoma* genome was 1.46, an extremely low value compared to that of the human genome (~2.1), suggesting that most mutations in *Branchiostoma* have occurred recently with a high mutation per generation. The high polymorphism rates and low Ts/Tv ratio of the whole genome confirm that *Branchiostoma* is one of the most genetically diverse species sequenced to date. Demographic inference analysis revealed that the effective population size of *B. belcheri* suffered from various degrees of reduction during the first two glacial periods (XG and NG) and remained at a very low level in other periods of the Quaternary. Thereafter, there was a remarkable population expansion of *B. belcheri* during the Greenlandian stage of the current geological epoch, termed the Holocene epoch. Mitochondria are important organelles in eukaryotic organisms; they can generate large quantities of energy in the form of ATP and play vital roles in many metabolism processes. We detected a total of 650 variations, including 415 SNPs and 235 Indels, in the *B. belcheri* mt genome, and further analyzed their genetic mutations. These findings could provide valuable information for further research into the function and evolution of the *Branchiostoma* mt genome. Furthermore, we identified all “probably damaging” and “possibly damaging” mutations in the phagocytic intracellular digestion-associated genes of *Branchiostoma* diverticulum epithelial cells; these mutations likely influence the capacity of *Branchiostoma* to phagocytize and digest food particles, such as algal cells. In the future, we will make full use of these genetic variations to investigate the phagocytic intracellular digestion of *Branchiostoma* and the genetic regulation of genotypes on phenotypes.

## Figures and Tables

**Table 1:** The genomic mapping results of 20 *B. belcheri* individuals sequenced in this study.

Sample	Raw reads^a^	Clean reads^b^	Mapped reads^c^	Mapped ratio (%)	Depth (X)	Polymorphism (%)
BB_Male1	44,114,632	43,885,024	42,243,757	96.26	13.04	3.07
BB_Male2	41,274,682	41,012,848	39,327,722	95.89	12.17	3.04
BB_Male3	43,130,836	42,897,930	41,053,672	95.70	12.69	3.12
BB_Male4	45,915,202	45,674,876	43,820,708	95.94	13.54	3.12
BB_Male5	49,463,716	49,233,046	47,271,411	96.02	14.61	3.18
BB_Male6	41,734,308	41,520,068	39,886,922	96.07	12.35	3.06
BB_Male7	37,587,766	37,362,618	35,821,114	95.87	11.08	2.93
BB_Male8	43,835,218	43,532,754	41,780,961	95.98	12.93	3.12
BB_Male9	40,378,470	40,158,296	38,450,579	95.75	11.91	3.02
BB_Male10	38,024,466	37,793,532	36,247,624	95.91	11.21	2.95
BB_Female1	40,737,494	40,462,332	38,851,551	96.02	12.02	2.97
BB_Female2	39,294,422	39,111,470	37,548,055	96.00	11.61	3.01
BB_Female3	45,074,570	44,876,004	42,927,757	95.66	13.25	3.09
BB_Female4	51,076,032	50,810,364	48,840,662	96.12	15.04	3.17
BB_Female5	37,747,132	37,611,242	35,960,749	95.61	11.01	2.67
BB_Female6	45,734,938	45,484,396	43,738,189	96.16	13.53	3.14
BB_Female7	37,719,652	37,478,310	35,922,565	95.85	11.11	2.95
BB_Female8	44,468,780	44,001,858	41,441,734	94.18	12.74	3.06
BB_Female9	38,781,426	38,557,444	36,974,687	95.90	11.45	2.97
BB_Female10	42,382,180	42,136,364	40,460,892	96.02	12.53	3.09
SRR1174914	—	210,710,342	195,932,673	92.99	42.30	3.69
Average^d^	42,423,796	42,180,039	40,428,566	95.85	12.49	3.04

^a^Raw sequencing reads without any filtration. ^b^Reads after removing low-quality reads under quality control criteria. ^c^Clean reads mapped to reference genome after removing secondary reads. ^d^Average of the above values excluding SRR1174914.

**Table 2:** Statistics of variations in the *B. belcheri* population.

Genomic region^a^		SNP	InDel	Total variation	Rate
*Nucleus*	*Total*	*37,589,099*	*14,541,374*	*52,130,473*	*100.00%*

*Exonic*		*4,412,877*	*224,658*	*4,637,535*	*8.90%*
	*Frameshift*	*—*	*134,185*	*134,185*	*0.26%*
	*Nonframeshift*	*—*	*78,739*	*78,739*	*0.15%*
	*Nonsynonymous*	*1,467,863*	*—*	*1,467,863*	*2.82%*
	*Synonymous*	*2,818,189*	*—*	*2,818,189*	*5.41%*
	*Stop codon gained*	*11,487*	*5,820*	*17,307*	*0.03%*
	*Stop codon loss*	*1,275*	*280*	*1,555*	*~0*
	*Unknown*	*114,063*	*5,634*	*119,697*	*0.23%*

*UTR 3′*		*1,173,085*	*480,925*	*1,654,010*	*3.17%*
*UTR 5′*		*386,621*	*112,512*	*499,133*	*0.96%*
*UTR 3′ and UTR 5′ ^b^*		*566*	*119*	*685*	*~0*
*Splicing*		*5,702*	*15,967*	*21,669*	*0.04%*
*Upstream (1 kb)*		*1,447,174*	*594,644*	*2,041,818*	*3.92%*
*Downstream (1 kb)*		*1,448,774*	*653,116*	*2,101,890*	*4.03%*
*Up- and downstream (1 kb)^c^*		*250,316*	*123,295*	*373,611*	*0.72%*
*NcRNA^d^*		*1,289,222*	*451,365*	*1,740,587*	*3.34%*
*Intronic*		*18,339,243*	*8,714,521*	*27,053,764*	*51.90%*
*Intergenic*		*8,835,519*	*3,170,252*	*12,005,771*	*23.03%*

**Mitochondrion**	**Total**	**415**	**235**	**650**	**100.00%**
	**Protein-coding genes**	**225**	**96**	**321**	**49.38%**
	**rRNA**	**138**	**94**	**232**	**35.69%**
	**tRNA**	**50**	**43**	**93**	**14.31%**
	**Intergenic**	**2**	**2**	**4**	**0.62%**

^a^Italic values and bold values represent variants in nucleus and mitochondrion, respectively. ^b^Variants located in both 5′ UTR and 3′ UTR regions (possibly for two different genes). ^c^Variants located in both downstream and upstream regions (possibly for two different genes). ^d^Variants located overlapping a transcript without coding annotation.

**Table 3:** Summary of heterozygosity and polymorphism in each *B. belcheri* individual.

Accession	Heterozygous	Homozygous	Total SNP	Total InDel	Total variations	Heterozygosity (%)	Polymorphism (%)
BB_Male1	7,236,365	5,669,342	10,039,198	2,866,509	12,905,707	1.67	3.07
BB_Male2	7,181,037	5,604,379	9,934,834	2,850,582	12,785,416	1.73	3.04
BB_Male3	7,448,022	5,675,415	10,181,479	2,941,958	13,123,437	1.75	3.12
BB_Male4	7,504,542	5,620,838	10,192,631	2,932,749	13,125,380	1.78	3.12
BB_Male5	7,672,392	5,701,874	10,365,510	3,008,756	13,374,266	1.68	3.18
BB_Male6	7,210,381	5,654,957	9,995,854	2,869,484	12,865,338	1.57	3.06
BB_Male7	6,738,319	5,590,704	9,603,984	2,725,039	12,329,023	1.73	2.93
BB_Male8	7,453,916	5,674,691	10,185,507	2,943,100	13,128,607	1.64	3.12
BB_Male9	7,056,636	5,627,169	9,859,911	2,823,894	12,683,805	1.58	3.02
BB_Male10	6,798,463	5,592,276	9,648,505	2,742,234	12,390,739	1.60	2.95
BB_Female1	6,878,788	5,611,338	9,703,990	2,786,136	12,490,126	1.62	2.97
BB_Female2	6,936,306	5,722,402	9,770,419	2,888,289	12,658,708	1.71	3.01
BB_Female3	7,350,721	5,649,596	10,091,242	2,909,075	13,000,317	1.78	3.09
BB_Female4	7,646,739	5,696,195	10,356,814	2,986,120	13,342,934	1.36	3.17
BB_Female5	5,837,390	5,412,573	8,831,037	2,418,926	11,249,963	1.75	2.67
BB_Female6	7,518,735	5,693,033	10,250,508	2,961,260	13,211,768	1.59	3.14
BB_Female7	6,822,458	5,591,591	9,663,883	2,750,166	12,414,049	1.68	2.95
BB_Female8	7,235,261	5,633,362	9,998,760	2,869,863	12,868,623	1.60	3.06
BB_Female9	6,882,518	5,602,731	9,708,333	2,776,916	12,485,249	1.71	2.97
BB_Female10	7,345,987	5,667,635	10,102,188	2,911,434	13,013,622	1.66	3.09
SRR1174914	9,750,311	5,976,946	12,001,160	3,726,097	15,727,257	2.29	3.69
Average^a^	7,137,749	5,634,605	9,924,229	2,848,125	12,772,354	1.66	3.04

^a^Average of the above values excluding SRR1174914.

**Table 4:** Variations in mitochondrial protein-coding genes of *B. belcheri*.

Gene	Start	End	Synonymous	Nonsynonymous	Frameshift	Nonframeshift	Ka/Ks
*CYTB*	1	1141	3	0	6	0	0
*ND1*	3712	4656	14	5	12	1	0.36
*ND2*	4856	5896	0	0	0	0	—
*COX1*	6217	7764	5	5	5	1	1
*COX2*	7907	8597	60	11	14	1	0.18
*ATP8*	8663	8827	0	2	7	6	—
*ATP6*	8821	9504	14	1	8	2	0.07
*COX3*	9504	10292	24	4	10	1	0.17
*ND3*	10293	10646	1	1	5	2	1
*ND4L*	10712	10987	0	0	0	0	—
*ND4*	10989	12347	24	5	9	0	0.21
*ND5*	12548	14344	29	17	5	3	0.59
*ND6*	14503	15006	0	0	0	0	—

**Table 5:** Prediction of functional effects of nonsynonymous variations in phagocytic intracellular digestion-associated genes.

Gene	EST cluster	Probably damaging	Possibly damaging	Benign	Unknown	nsSNP rate
Cathepsin L^ab^	433	12	28	247	0	3.79%
Cathepsin B^ab^	131	1	3	5	0	0.86%
Cathepsin D^ab^	11	0	0	1	2	0.26%
Cathepsin Z^ab^	12	2	4	13	1	2.17%
Ferritin^b^	505	3	6	26	0	2.12%
Trypsin-like serine protease^a^	300	25	52	156	1	2.74%
Lysozyme-like^ac^	2	5	10	18	17	7.75%
Lysozyme C^ac^	92	2	3	15	8	2.23%
Lysozyme G^ac^	19	1	2	6	0	1.12%
Pancreatic lipase-like protein^a^	108	12	41	98	24	1.66%
VCBP1^c^	11	3	8	15	2	2.78%
VCBP3^c^	6	1	4	10	0	1.50%
VCBP4^c^	58	12	17	44	0	7.03%
VCBP5^c^	4	38	33	68	0	12.91%
Carboxypeptidase Z/N^a^	74	4	12	32	0	1.69%
Tetraspanin^c^	58	11	32	94	0	2.01%
Legumain^ab^	47	2	0	9	0	0.84%
Saposin B^a^	43	2	1	3	0	0.77%
Subtilisin-like protease (proteinase T)^a^	42	2	3	7	9	1.72%
Arylsulfatase B^a^	36	18	45	176	0	2.73%
Gram-negative bacteria-binding protein^c^	28	6	7	23	2	1.85%
Endo-beta-1,4-glucanase^a^	25	3	11	14	0	0.56%
Alpha2-macroglobulin^c^	18	5	22	40	2	1.53%
Methionine adenosyltransferase^b^	18	1	4	27	0	2.72%
Plasminogen^a^	16	11	19	34	16	4.08%
Chitotriosidase 1-like protein^ac^	27	25	52	147	6	3.34%
Big defensin^c^	7	1	6	4	1	1.99%
Peroxiredoxin V^b^	5	2	0	6	0	1.41%
Proprotein convertase subtilisin/kexin type 1^a^	5	4	22	48	3	3.25%
Toll-interacting protein^c^	1	0	0	1	0	0.12%

^a^Digestive or hydrolytic genes. ^b^Genes concerned with immune reactions. ^c^Typical immune genes.

## Data Availability

All sequencing data for the twenty *Branchiostoma* accessions have been submitted to the NCBI Short Read Archive (SRA) under the BioProject accession: PRJNA510004 (accession number: SRR832468-SRR8324701). The high-depth sequencing data from Zhanjiang used in this study were downloaded from NCBI SRA database under the accession number SRR1174914. Supporting data (Raw variant sets, filtered variant sets and variant annotations) can be downloaded from (http://bio.njfu.edu.cn/bbr/Downloads/).

## Supplementary Materials

Supplementary 1 Table S1: The genomic mapping results of all *B. belcheri* individuals in this study.

Supplementary 2 Table S2: Transitions and transversions in each *B. belcheri* individual.

Supplementary 3 Table S3: Comparison of polymorphism rates between each *B. belcheri* individual.

Supplementary 4 Table S4: Comparison of variations in different regions of *B. belcheri* genome.

Supplementary 5 Table S5: Prediction of functional effects of nonsynonymous SNPs in selected housekeeping and immune genes.

Supplementary 6 Table S6: Summary of damage variations in phagocytic intracellular digestion associated genes.
